# A European survey of older peoples’ preferences, and perceived barriers and facilitators to inform development of a medication-related fall-prevention patient portal

**DOI:** 10.1007/s41999-024-00951-w

**Published:** 2024-04-08

**Authors:** Kim J. Ploegmakers, A. J. Linn, S. Medlock, L. J. Seppälä, G. Bahat, M. A. Caballero-Mora, B. Ilhan, F. Landi, T. Masud, Y. Morrissey, J. Ryg, E. Topinkova, N. van der Velde, J. C. M. van Weert, European Geriatric Medicine Society (EuGMS) Task and Finish group on Fall-Risk Increasing Drugs (FRIDs)

**Affiliations:** 1 Section of Geriatric Medicine, Department of Internal Medicine, Academic Medical Center, Amsterdam UMC, University of Amsterdam, D3-227, Meibergdreef 9, 1105 AZ Amsterdam, The Netherlands; 2 Amsterdam Public Health Research Institute, Amsterdam, The Netherlands; 3 Amsterdam School of Communication Research/ASCoR, University of Amsterdam, Amsterdam, The Netherlands; 4 Department of Medical Informatics, Amsterdam UMC Location University of Amsterdam, Meibergdreef 9, Amsterdam, The Netherlands; 5 Division of Geriatrics, Department of Internal Medicine, Istanbul Medical Faculty, Istanbul University, Istanbul, Turkey; 6 Servicio de Geriatría, Hospital General Universitario de Ciudad Real, Ciudad Real, Spain; 7 Division of Geriatrics, Department of Internal Medicine, Liv Hospital Vadistanbul, Istanbul, Turkey; 8 Fondazione Policlinico Universitario A. Gemelli IRCCS, Catholic University, Rome, Italy; 9 Nottingham University Hospitals NHS Trust, Nottingham, UK; 10 Health Care of Older People, East Kent Hospitals University NHS Foundation Trust, Canterbury, Kent UK; 11 Department of Geriatric Medicine, Odense University Hospital, Odense, Denmark; 12 Geriatric Research Unit, Department of Clinical Research, University of Southern Denmark, Odense, Denmark; 13 Department of Geriatrics and Gerontology, 1st Faculty of Medicine, Charles University, Prague, Czech Republic; 14 Faculty of Health and Social Sciences, South Bohemian University, České Budějovice, Czech Republic

**Keywords:** Patient Portal, Barriers, Facilitators, Falls prevention, Older people

## Abstract

**Purpose:**

Falls are a major and growing health care problem in older adults. A patient portal has the potential to provide older adults with fall-prevention advice to reduce fall-risk. However, to date, the needs and preferences regarding a patient portal in older people who have experienced falls have not been explored. This study assesses content preferences, potential barriers and facilitators with regard to using a patient portal, as perceived by older people who have experienced falls, and explores regional differences between European participants.

**Methods:**

We conducted a survey of older adults attending an outpatient clinic due to a fall or fall-related injury, to explore their content preferences, perceived barriers, and facilitators with respect to a fall-prevention patient portal. Older adults (*N* = 121, 69.4% female, mean age: 77.9) were recruited from seven European countries.

**Results:**

Almost two-thirds of respondents indicated they would use a fall-prevention patient portal. The portal would preferably include information on Fall-Risk-Increasing Drugs (FRIDs), and ways to manage other related/relevant medical conditions. Facilitators included a user-friendly portal, with easily accessible information and physician recommendations to use the portal. The most-commonly-selected barriers were privacy issues and usage fees. A family member’s recommendation to use the portal was seemingly more important for Southern and Eastern European participants compared to the other regions.

**Conclusion:**

The majority of older people with lived falls experience expressed an interest in a fall-prevention patient portal providing personalized treatment advice to prevent further falls. The results will be used to inform the development of a fall-prevention patient portal. The fall-prevention patient portal is intended to be used in addition to a consultation with a physician. Future research is needed to explore how to prevent falls in older patients who are not interested in a fall-prevention patient portal.

**Supplementary Information:**

The online version contains supplementary material available at 10.1007/s41999-024-00951-w.

## Key summary points

**Aim:**

To explore content preferences, potential barriers and facilitators as perceived by European older adults who have experienced falls with regards to using a fall-prevention patient portal, and to explore regional differences between European participants.

**Findings:**

About two-thirds of the participants (*n* = 121) reported interest in a fall-prevention patient portal providing information on risk factors for falls, relevant medical conditions, Fall-Risk Increasing Drugs (FRIDs), and advice on how to manage fall-related conditions. Fees for use and privacy concerns appeared to be the most important barriers, while a user-friendly portal with easily-accessible information and a physician recommendation seemed to be the most important facilitators. A recommendation for portal use by a family member appeared to be a more important facilitator for participants from Southern and Eastern Europe compared to the other regions.

**Message:**

There is considerable interest in a fall-prevention patient portal providing personalized treatment advice, used in addition to a consultation with a physician. The barriers and facilitators to using a portal as identified by participants should be taken into account when developing future fall-prevention patient portals in order to optimize clinical effectiveness.

**Supplementary Information:**

The online version contains supplementary material available at 10.1007/s41999-024-00951-w.

## Introduction

Falls occur frequently in older adults (≥ 65 years). They can result in fractures, other injuries and hospital admission, ultimately leading to reduced quality of life, and potentially to increased mortality [1, 2]. In financial terms, falls are amongst the top 20 most expensive medical conditions in older adults [3, 4]. With current rapid population aging [5], fall-related incidents and resultant health care costs are likely to increase, making effective fall prevention ever more pressing.

An important risk factor for falls is the use of Fall-Risk-Increasing Drugs (FRIDs) [6–8]. FRID deprescribing is effective in reducing fall risk as a part of a multi-domain, fall-prevention intervention [9] and has been recommended in the recent World Falls Guidelines [10]. However, the process of deprescribing FRIDs can be challenging to implement in clinical practice [11]. This is partly due to patient-related factors. Although older adults are often FRID users [12], they are generally unaware that falls can be caused by their medication [13].

They may consider falls as something to be expected as part of the ageing process, thus something inevitable rather than a preventable medical condition [14].

A patient portal has the potential to reduce older adults' fall risk by informing patients about FRIDs and other fall risk factors. A patient portal is a personal, user-friendly, secure computer website that allows patients to access their own personal health information, for example their hospital discharge summaries or medication lists, in a convenient way at anytime and anywhere with internet access. Some patient portals enable patients to message their physician, order repeat prescriptions, or view educational materials about (for example) how to reduce fall risk [15]. Previous studies have shown that the use of patient portals can promote information sharing between patients and physicians. This has the potential to empower patients in shared decision-making, by enabling them to better express their ideas, views and concerns about their treatment plans. This engagement can encourage active participation in self-care and self-management which is crucial in the effective prevention of falls [16, 17].

Despite the considerable potential of patient portals, older adults exhibit a tendency to underutilize them [18]. A recent review identified several barriers and facilitators regarding the use of patient portals from the perspective of older adults [19]. According to this review, this underutilization may because of a lack of computer and internet access and/or the skills needed to use digital health technologies in this age group [19]. Among European older adults, 61% report using the internet in the past 3 months [20]. Internet usage is less among the oldest old (80+) compared to younger older adults (65–75 years) [21]. Additionally, the use of a patient portal appears to be influenced by age and educational level, whereas older and lower educated people tend to use a portal less than younger, highly educated people [17]. When older adults do use a patient portal, they do so mainly to view test results [22].

In the context of fall prevention, it is not known if there is a specific need for a fall-prevention patient portal to assist informed decision making to reduce fall risk, and, if so, which type or format of information would be most helpful to older adults to support them in their decision making. Therefore, we aimed to explore the preferred content of a patient portal and perceived barriers and facilitators with respect to the use of a fall-prevention patient portal from the perspective of older adults with lived falls experience. This study is part of the AD*F*ICE_IT project, which aims to optimize FRID deprescribing in older adults with a clinical decision support system and patient portal to reduce fall risk (for study protocol see [24]). Potential end-users of the AD*F*ICE_IT portal are older adults (65+) with a fall in the past year, a Mini-Mental State Examination (MMSE) of 21 or higher and who use at least one FRID. Although we recognize that there are other important fall risk factors besides FRIDs, such as physical function, or environmental or behavior aspects [23], the scope of this survey and overarching project (AD*F*ICE_IT) is optimizing deprescribing aimed at falls. The results of this survey will be used to guide the development of a patient portal. It also remains unknown whether these patient preferences are uniform across European countries or if variations exist, like the variations in population computer skills and/or internet among the different European countries. We hypothesized that regional differences (for example in computer and internet access) could affect participants’ preferences, and their perceptions of the barriers and facilitators with regard to the use of a patient portal. Therefore, we aimed to explore European regional differences in preferences, barriers and facilitators for portal use.

## Methods

### Survey development

The survey questionnaire was developed specifically for this study. An initial draft was composed in English by two authors (KP and AL) and sent to all European FRIDs Task and Finish Group members to obtain their comments and feedback. Two feedback rounds were required to achieve consensus on the questions to be included in the survey which was then further linguistically checked by a participating native English speaker (YM). From the English version, the survey was subsequently translated into other European languages by native speakers in the group including Czech (ET), Danish (JR), Dutch (KP and NV), Italian (CRC, FL), Spanish (AB, MACM) and Turkish (GB and BI). The final English version of the survey can be found in Supplement 1.

### Survey questions

The survey consisted of five sections: patient characteristics, previous experience with and intent to use a patient portal, preferred patient portal content, barriers and facilitators. The aim of the survey was to inform the development of a fall-prevention patient portal and assess patient knowledge of risk factors for medication-related falls.

#### Patient characteristics

The survey contained questions on patient characteristics including demographics (e.g. age, gender, educational level, social circumstances), prescribed polypharmacy (defined as using five or more medications), history of falls, and general health. The educational levels listed were localized per country. The survey also contained eight statements about participants’ computer and internet skills, rated on a five-point scale.

#### Previous experience with and intent to use a patient portal

Participants were asked if they had ever used a patient portal (yes/no), and whether they would like to use a fall-prevention portal and if they would like to know their medication-related fall risk (yes/no/maybe). Participants who answered "no" or "maybe" on the latter two questions were directed to answer additional clarifying questions. Participants’ previous experience of using a patient portal, intent to use a fall-prevention patient portal and to know their fall-risk estimation were measured categorically (yes, no, maybe). The definitions of a patient portal as stated in the introduction and of a fall (an unexpected event in which the participants come to rest on the ground, floor, or lower level [26]) were provided in the survey.

#### Preferred content features

To ascertain the patient portal features and topics of information older patients would find most helpful, two authors (KP and AL) drafted an initial list of potential features. This list was developed by analyzing features present in a patient portal that is currently used in 13% of Dutch (University) hospitals (Epic/MyChart). Additionally, the analysis considered fall-prevention patient information leaflets and exercise programs currently in use. This resulted in a final list of* n* = 19 features. Participants could select as many of the 19 listed features as they believed a patient portal should ideally contain. If a preferred feature was not included in the provided options, participants were given the opportunity to add it in an open text box.

#### Preferred barriers and facilitators

To assess barriers and facilitators to using a fall patient portal as perceived by older adults with lived falls experience, an initial list comprising eight barriers and ten facilitators was compiled. This list drew upon insights from a systematic review [19] and the Technology Acceptance Model [27]. The goal of these items was to gain insight into possible usability challenges associated with poor portal use. Supplement 2 provides an overview of the sources informing the selection of barriers and facilitators. Participants were asked to select aspects and circumstances that might deter them from using a patient portal (barriers) and aspects and circumstances that encourage them to use a portal (facilitators). Participants were asked to select a maximum of four barriers and a maximum of five facilitators that best applied to them. If participants felt there were barriers or facilitators missing from the list, they were able to add these in an open text box.

### Data collection

The survey was conducted by members of the European Geriatric Medicine Society (EuGMS) Task and Finish Group on FRIDs. Thirteen members of the Task and Finish Group were approached and informed about the study. Seven European countries participated. Participating members were asked to recruit eligible patients who were adults aged 65 and over who had consulted a health care professional due to a fall or fall-related injury during the previous year. Participants were recruited in the outpatient clinics of the participating centers.

The survey was conducted between January and July 2019 in seven European nations: the Czech Republic, Denmark, Italy, the Netherlands, Spain, Türkiye, and the United Kingdom. Older adults with lived falls experience attending an outpatient clinic in the local hospital of each participating Task and Finish group member were invited to participate. Each Task and Finish group member was asked to recruit a minimum of 15 patients and was given approximately 3 months to submit completed patient surveys. Participants could fill in the survey either on paper or online. LimeSurvey was used for the online survey. It was left up to the participating center to decide if they would offer it on paper, online, or both. Participants could fill in the survey by themselves or with help of family member or a research assistant if preferred.

### Analysis

Participating countries were categorized into four regions as designated by United Nations geographical definitions [28] as follows: Denmark and the United Kingdom were classified as Northern Europe, the Netherlands as Western Europe, Spain and Italy as Southern Europe, and Czech Republic and Türkiye as Eastern Europe. Participants’ educational levels were coded in accordance with International Standard Classification of Education (ISCED) levels [29]. ISCED levels 0–2 were recoded as “Low” Educational level, levels 3 and 4 as "Average”, and ISCED level 5–8 as “High” Educational level.

To compare differences between European regions, we used chi square tests for categorical variables and Multivariate Analysis of Variance (MANOVA) for continuous variables. A p-value of 0.05 or lower was considered statistically significant. Participants who checked zero boxes for the preferred features, barrier, or facilitator question were excluded from these analyses. All data were analyzed with SPSS for Windows version 26.0.0.1 (IBM Corp., New York).

Open text box entries were translated into English using an automated translation program. If answers were unclear, native speakers on our expert panel were contacted regarding the translation. Open text box entries were open-coded and classified by the first author (KP). In cases of uncertainty a second author (AL or SM) was consulted.

### Ethical approval

The Medical Ethical Committee of the Academic Medical Centre of the University of Amsterdam reviewed this study and ruled that no ethical approval was required (W18_285#18.331). All participants were asked to give written, informed consent.

## Results

### Study population

The survey was filled in by 125 participants from 7 European countries. Three participants did not meet the inclusion criteria and were, therefore, excluded from the analysis. All Spanish participants filled in the survey online, while the participants from all the other countries filled in the paper version of the survey. Table 1 shows participants’ baseline characteristics. In summary, participants had a mean (standard deviation, SD) age of 77.7 (7.9) years, 69.4% were female, and 48.7% had a low educational level. Overall, about half (46.7%) of the participants had internet access at home. Patient internet access and computer skills were significantly better in Western Europe and generally less advanced in Northern Europe.

**Table 1:** Participant characteristics

	Europe (*n* = 122)	Northern (*n* = 29)	Western (*n* = 42)	Southern (*n* = 20)	Eastern (*n* = 31)
Age (years), mean (SD), *n* = 120	77.7 (7.9)	80.4 (8.1)	75.8 (6.9)	80.5 (9.2)	76.1 (6.2)
Female	69.4%	72.4%	69.0%	65.0%	70.0%
Educational Level, *n* = 119					
Low	48.7%	57.7%	57.1%	35.0%	38.7%
Average	17.6%	11.5%	23.8%	25.0%	9.7%
High	33.6%	30.8%	19.0%	40.0%	51.6%
Living situation					
Own home independently	63.9%	58.6%	69.0%	35.0%*	80.6%
Own home with help	32.8%	41.4%	31.0%	45.0%	19.4%
Nursing home	3.3%	0	0	20.0%*	0
General health^a^, mean (SD), *n* = 120	6.3 (1.7)	6.1 (1.8)	6.5 (1.5)	6.1 (2.0)	6.3 (1.8)
Polypharmacy (yes), *n* = 121	57.9%	65.5%	61.9%	31.6%	61.3%
Walking aid use (yes), *n* = 121	55.4%	72.4%	45.2%	57.9%	51.6%
Internet access, *n* = 120					
At home	46.7%	25.0%	73.8%*	26.3%	41.9%
With help of others	19.2%	17.9%	16.7%	31.6%	16.1%
Yes, but not at home	1.7%	3.6%	2.4%	0	0
No	32.5%	53.6%	7.1%*	42.1%	41.9%
Feeling familiar with using a computer^b^, median (IQR), *n* = 116	2.5 (1–5)	1.0 (1–3.5)^W^	4.0 (2–5)^N^	2.0 (2–4)	2.0 (1–5)
Needing help from others using internet^b^, mean (SD), *n* = 117	4.0 (1–5)	4.0 (1–5)	2.0 (1–5)	4.0 (2–5)	4.0 (1–5)
Physical limitation preventing use of computer^b^, median (IQR), *n* = 116	1.5 (1–3)	2.0 (1–4.5)	1.0 (1–2)	2.0 (2–4)	2.0 (1–5)
Aware medication can cause falls? (yes), *n* = 120	49.2%	53.6%	54.8%	40.0%	43.3%
How did you learn this?, *n* = 62					
Physician	45.2%	52.6%	40.9%	75.0%	23.1%
Someone else told me	6.5%	15.8%	4.5%	0	0
Read it in leaflet	19.4%	10.5%	9.1%	0	61.5%*
Read it on internet	3.2%	5.3%	0	0	7.7%
Other	25.8%	15.8%	45.5%	25.0%	7.7%

*n* number of participants, *SD* standard deviation, *IQR* interquartile range

^a^Participants could score this item on a scale from 1 (very bad) to 10 (very good)

^b^Participants could score this item on a 5 point Likert scale, ranging from not applicable to me at all (1) to strongly applicable to me (5)

*p < 0.05. Northern Europe: Denmark and the United Kingdom; Western Europe: the Netherlands; Southern Europe: Italy and Spain; Eastern Europe: Czech Republic and Türkiye

About half of the participants (49.2%) were aware that falls can be caused by certain medications. Overall, they got this information most often from their physician, whereas Eastern Europeans got this information significantly more often from reading a leaflet. In the open text box, four participants stated that they logically reasoned this themselves based on the working mechanism and side-effects of some of their medications. Others learned about this through their former profession in health care (*n* = 3).

### Experience with and intention to use a fall-prevention patient portal with personalized fall risk

**Fig. 1:**
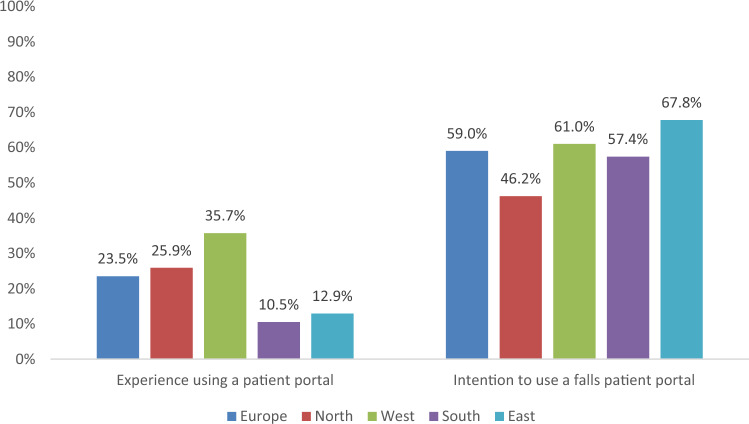
Patient portal use. Figure displays the number of participants responding with “yes” when asked if they have experience using a patient portal (left panel) and “yes” or “maybe” when asked if they would use a fall-prevention patient portal (right panel). Northern Europe: Denmark and the United Kingdom; Western Europe: the Netherlands; Southern Europe: Italy and Spain; Eastern Europe: Czech Republic and Türkiye

Figure 1 shows participants’ experience of using a patient portal and intention to use one.

Despite only a quarter of the participants having had experience of using a patient portal, around 60% of all participants indicated they would want to use, or would consider using one. Participants that indicated they would not or might not use a portal were asked to provide reasons. Of these (*n* = 63), reasons for not wanting to use a patient portal included not having internet access (47.6%) and anticipated difficulty using it (38.1%). In general, participants felt moderately confident about being able to use a patient portal (mean (SD) 3.2 (1.4) out of 5). Furthermore, over half the participants preferred in-person contact with their doctor compared to using a portal (52.4%).

Participants with home internet access had significantly more experience using a patient portal (46.4%) and were more inclined to use one (70.9%) than participants who did not have internet access (2.6% and 16.2%, respectively) (Supplement 3). No regional differences were observed.

### Features, barriers, and facilitators for a fall-prevention patient portal

#### Preferred content features of a patient portal

Table 2 shows the features that older people with lived falls experience would prefer a patient portal to contain, according to geographical region. Thirteen participants did not check any feature and were therefore excluded from the analysis of this section. These participants mainly came from Northern Europe (Supplement 4). The most-frequently-chosen features were: information on patients’ illnesses, FRIDs, and ways to manage fall-related conditions (for example dizziness or orthostatic hypotension). Features that were chosen the least were: experiences of other patients, useful links to other websites, and a dictionary of medical jargon. The feature “information about my illnesses” was chosen more often by participants from Eastern Europe than other regions. Southern European participants checked information on FRIDs less often than Western and Eastern European participants and information on fall prevention less than Eastern Europeans. Northern European participants perceived “access to their medical record” as a less important feature compared to Western European participants.

**Table 2:** Preferred features for a patient portal according to Geographical region

	Europe (*n* = 108)	Northern (*n* = 20)	Western (*n* = 38)	Southern (*n* = 19)	Eastern (*n* = 31)
	Mean (SD)	Mean (SD)	Mean (SD)	Mean (SD)	Mean (SD)
Information on my illness	0.56 (0.50)	0.55 (0.51)	0.50 (0.51)	0.37 (0.50)^E^	0.77 (0.43)^S^
Information on FRIDs	0.55 (0.50)	0.50 (0.51)	0.63 (0.49)^S^	0.26 (0.45)^W,E^	0.65 (0.49)^S^
Information on managing fall-related conditions	0.52 (0.50)	0.50 (0.51)	0.47 (0.51)	0.53 (0.51)	0.58 (0.50)
Information on falls	0.51 (0.50)	0.60 (0.50)	0.47 (0.51)	0.26 (0.45)	0.65 (0.49)
Access to my test results	0.50 (0.50)	0.40 (0.50)	0.61 (0.50)	0.26 (0.45)	0.58 (0.50)
Access to my medical record	0.50 (0.50)	0.25 (0.44)^W^	0.63 (0.49)^N^	0.37 (0.50)	0.58 (0.50)
Fall-prevention information	0.49 (0.50)	0.60 (0.50)	0.47 (0.51)	0.21 (0.42)^E^	0.61 (0.50)^S^
Physical exercises to prevent falls	0.46 (0.50)	0.60 (0.50)	0.47 (0.51)	0.21 (0.42)	0.52 (0.51)
Appointment reminder	0.44 (0.50)	0.35 (0.49)	0.47 (0.51)	0.32 (0.48)	0.52 (0.51)
Information on medication interactions	0.44 (0.50)	0.30 (0.47)	0.37 (0.49)	0.47 (0.51)	0.58 (0.50)
Physical exercises to stay healthy	0.43 (0.50)	0.60 (0.50)	0.34 (0.48)	0.37 (0.50)	0.45 (0.51)
Information on how I can improve my health in general	0.41 (0.49)	0.45 (0.51)	0.32 (0.47)	0.32 (0.48)	0.55 (0.51)
Order a repeat prescription	0.38 (0.49)	0.20 (0.41)	0.45 (0.50)	0.26 (0.45)	0.48 (0.51)
Access to my hospital discharge letter/ clinic letter	0.37 (0.49)	0.35 (0.49)	0.26 (.045)	0.32 (0.48)	0.55 (0.51)
Email communication with my physician	0.37 (0.49)	0.15 (0.37)	0.39 (0.50)	0.42 (0.51)	0.45 (0.51)
Ability to print information	0.32 (0.47)	0.25 (0.44)	0.34 (0.48)	0.26 (0.45)	0.39 (0.50)
Experiences of other patients with the same illness as mine	0.23 (0.42)	0.20 (0.41)	0.13 (0.34)^E^	0.16 (0.37)	0.42 (0.50)^W^
Useful links to other health websites	0.21 (0.41)	0.15 (0.37)	0.11 (0.31)	0.26 (0.45)	0.35 (0.49)
Dictionary of medical jargon	0.20 (0.40)	0.15 (0.37)	0.21 (0.41)	0.11 (0.32)	0.29 (0.46)

*n* number of participants, *FRIDs* fall-risk increasing drugs

Features are ranked from most chosen to least chosen based on the European percentage. Letters in superscript indicate a significant difference between regions. For example, S^E^ means that the Southern region is significantly different from Eastern Europe

Northern Europe: Denmark and the United Kingdom; Western Europe: the Netherlands; Southern Europe: Italy and Spain; Eastern Europe: Czech Republic and Türkiye

#### Medication-related fall-risk estimation

**Fig. 2:**
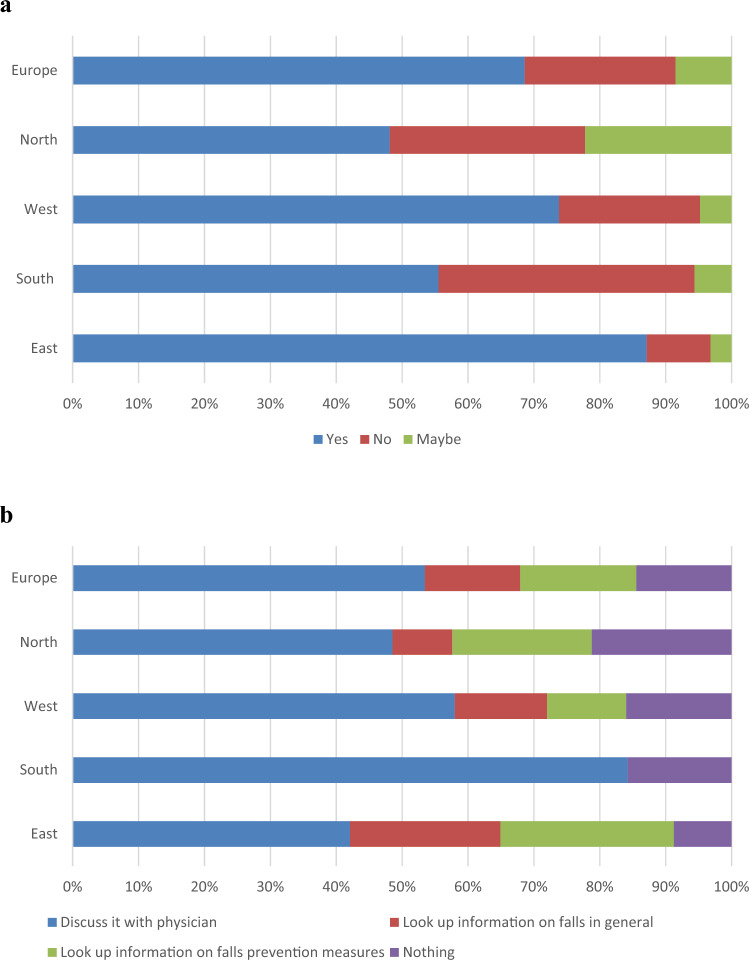
Figure displays participants’ interest in their personalized fall risk (**a**) and what participants would do with this information (**b**). Northern Europe: Denmark and the United Kingdom; Western Europe: the Netherlands; Southern Europe: Italy and Spain; Eastern Europe: Czech Republic and Türkiye

Figure 2 shows participants’ interest in a personalized medication-related fall-risk estimate.

Nearly 70% of participants would want to know their medication-related fall risk. The preference to know a personalized fall-risk estimate was not influenced by internet access (Supplement 3).

Most participants (70.2%) would want to discuss their fall-risk estimate with their physician. Furthermore, some participants would look up information about falls in general (19.0%) and information on fall-prevention measures (23.0%). Knowing their fall risk would encourage some patients to be more careful (*n* = 4, open text box entry). Participants that indicated they would not or might not want to know their fall risk were asked to provide reasons. Of these (*n* = 37), reasons for patients not wanting to know their fall-risk estimate included that they did not consider it relevant (*n* = 13) and some participants stated that it would “scare” them to know their fall risk estimate (*n* = 13). A few participants stated that their fall risk had already been reduced, because FRIDs had been stopped (*n* = 2). Some said that withdrawal of FRIDs was not possible in their case, because they needed the medication for their health conditions (*n* = 2). Therefore, these patients reported no interest in knowing their personalized fall-risk estimate.

#### Barriers and facilitators

Table 3 shows the barriers to using a patient portal and Table 4 shows facilitators of patient portal usage as identified by patients according to European region. At least one barrier was reported by 102 of the 122 participants, and 96 participants filled in at least 1 facilitator. Participants with missing data came mainly from Northern Europe (Supplement 4). Nine participants stated that they had no interest in the patient portal and six stated that they did not have a computer or internet access and, therefore, they did not fill in the questions on barriers and facilitators. A total of 33 participants filled in the open text entries.

**Table 3:** Barriers for using a fall-prevention patient portal

	Europe (*n* = 102)	Northern (*n* = 19)	Western (*n* = 36)	Southern (*n* = 19)	Eastern (*n* = 28)
	Mean (SD)	Mean (SD)	Mean (SD)	Mean (SD)	Mean (SD)
Paying for usage	0.65 (0.48)	0.42 (0.51)	0.75 (0.44)	0.53 (0.51)	0.75 (0.44)
Privacy issues	0.61 (0.49)	0.53 (0.51)	0.64 (0.49)	0.68 (0.48)	0.57 (0.50)
Doesn’t improve my health	0.31 (0.47)	0.32 (0.48)	0.22 (0.42)	0.32 (0.48)	0.43 (0.50)
Only available online	0.31 (0.47)	0.42 (0.51)	0.19 (0.40)	0.53 (0.51)	0.25 (0.44)
Slow response from doctor	0.31 (0.47)	0.21 (0.42)	0.39 (0.49)	0.32 (0.48)	0.29 (0.46)
Can’t communicate with doctor	0.31 (0.47)	0.37 (0.50)	0.17 (0.38)	0.47 (0.51)	0.36 (0.49)
Illustrations difficult to comprehend	0.27 (0.45)	0.11(0.32)^E^	0.19 (0.40)	0.32 (0.48)	0.46 (0.51)^N^
Difficult to enter text	0.24 (0.43)	0.21 (0.42)	0.14 (0.35)	0.42 (0.51)	0.25 (0.44)

Data are presented as percentage of participants selecting a barrier (rank of the barrier based on number of participants selecting a barrier in the region)

*n* number of participants, *SD* standard deviation

Letters in superscript indicate a significant difference between regions. For example, N^E^ means that the Northern region is significantly different from Eastern Europe. Northern Europe: Denmark and the United Kingdom; Western Europe: the Netherlands; Southern Europe: Italy and Spain; Eastern Europe: Czech Republic and Türkiye

**Table 4:** Facilitators for using a fall-prevention patient portal

	Europe (*n* = 102)	Northern (*n* = 19)	Western (*n* = 36)	Southern (*n* = 19)	Eastern (*n* = 28)
	Mean (SD)	Mean (SD)	Mean (SD)	Mean (SD)	Mean (SD)
Easy to use	0.66 (0.48)	0.67 (0.49)	0.60 (0.50)	0.63 (0.50)	0.74 (0.45)
Easy to find information	0.52 (0.50)	0.33 (0.49)	0.57 (0.50)	0.47 (0.51)	0.59 (0.50)
Use recommended by my doctor	0.51 (0.50)	0.33(0.49)	0.54 (0.51)	0.47 (0.51)	0.59 (0.50)
Can share my medical information with my doctor	0.43 (0.50)	0.27 (0.46)	0.51 (0.51)	0.26 (0.45)	0.52 (0.51)
Support from nurse	0.32 (0.47)	0.53 (0.52)	0.31 (0.47)	0.16 (0.37)	0.33 (0.48)
Additional info on my illness and health	0.31 (0.47)	0.33 (0.49)	0.20 (0.41)	0.37 (0.50)	0.41 (0.50)
Video explaining portal	0.30 (0.46)	0.13 (0.35)	0.26 (0.44)	0.21 (0.42)	0.52 (0.51)
Use recommended by my family	0.27 (0.45)	0.13 (0.35)	0.09 (0.28)^S,E^	0.42 (0.51)^W^	0.48 (0.51)^W^
Voice commands	0.22 (0.42)	0.20 (0.41)	0.09 (0.28)^S^	0.47 (0.51)^W^	0.22 (0.42)
Written info accompanied by illustrations	0.21 (0.41)	0.13 (0.35)	0.09 (0.28)^S^	0.42 (0.51)^W^	0.26 (0.45)

Data are presented as percentage of participants selecting a barrier (rank of the barrier based on number of participants selecting a barrier in the region)

*n* number of participants

Letters in superscript indicate a significant difference between regions. For example, W^S,E^ means that the Western region is significantly different from Southern and Eastern Europe. Northern Europe: Denmark and the United Kingdom; Western Europe: the Netherlands; Southern Europe: Italy and Spain; Eastern Europe: Czech Republic and Türkiye

Barriers most often selected were: paying for a patient portal, privacy issues, and a portal that does not improve a patients’ health. Difficulty entering text was perceived as a barrier more often in the East than in other regions.

Additional barriers suggested in the open entry section included having no internet or computer (*n* = 6). Small numbers of patients stated other preferences: two participants felt that a patient portal was only for young people, two participants stated that they would rather discuss their medical problems face to face with their physician (*n* = 2) and one stated that they trusted in the knowledge and information provided by their doctor (*n* = 1).

The facilitators selected most often were: a patient portal that is easy to use, a portal on which information is easy to find, and a portal that is recommended by a patient’s own physician.

In the South and the East, perceiving that family members recommended patient portal use was more often a facilitating factor than in the North and West. A patient portal with voice commands and written information that is accompanied by illustrations were perceived as facilitators more often by Southern Europeans compared to Western Europeans.

Additional facilitators that were entered in the open entry section included: a portal without passwords (*n* = 2), clear information and information on what the patient can do himself or herself to prevent falls (*n* = 3), personalized advice and treatment plans which included a clear explanation about a proposed treatment or procedure (*n* = 3), and a list of doctors’ appointments (*n* = 1). Also, patients wanted the patient portal to be linked to other existing patient portals and accessible to relatives and other health care professionals such as Home care nurses (*n* = 2).

## Discussion

With a European survey, we explored features, barriers and facilitators for a fall-prevention patient portal using a survey among older European adults with lived fall experience. About two-thirds of participants reported interest in a fall-prevention patient portal providing information on risk factors for falls, relevant medical conditions, Fall-Risk Increasing Drugs (FRIDs), and advice on ways to manage fall-related conditions. A fee for use and privacy concerns were the most important barriers, while a user-friendly portal with easily accessible information and physician recommendations were the most important facilitators. Differences between European regions were found for anticipated usage, barriers and facilitators and information preferences.

### Preferred content features to inform portal development

The results showed that about half of the participants were aware that medication can cause falls and approximately 70% of participants were interested in knowing their personal fall risk. Our results also showed that the majority of older adults with lived fall experience felt a need for fall prevention information such as medication-related fall-risk estimates and information on their conditions, FRIDs, and ways to manage fall-related conditions. As well as the goal of preventing falls, the role of fall-prevention programs in conveying lifestyle advice to improve health in general and maintain independence should also be emphasized to help older adults to keep motivated to continue with fall prevention programs [30]. When developing a patient portal for older adults, a user-centered design (UCD) approach is advised to align the design of a patient portal to the preferences of older fallers. UCD is defined as a framework for a design process that increases the usability and acceptance of a system [31]. The focus of the UCD approach is to engage end-users in the design process to tailor the portal to them, to address their needs, abilities and characteristics [32]. This is especially important considering the age range of participants—a 60-year-old and an 80-year-old are likely to have very heterogenous preferences and skills regarding technology [33]. Utilizing UCD can break the cycle of technological development that excludes older persons and pave the way for an inclusive development process that actively involves them. This may contribute to the development of successful and adoptable patient portals for older people [31]. This study on European content preferences, facilitators, and barriers to using a patient portal represents a first effort to develop a patient portal that resonates with its users. By delving into the specific needs and challenges faced by European older adults, the study is able to inform the development of a patient portal that aligns closely with user expectations and experiences and lays the foundation for the creation of a more widely accepted and user-friendly patient portal within the European context.

### Providing information on fall risk

A patient portal was perceived by 60% of the participants to be an acceptable vehicle for delivering information on fall risk. According to our participants, the portal would ideally be used in addition to a consultation with their physician. Providing patients with some preparatory information before or after the consultation may result in patients processing fall-risk information more thoroughly, thus enabling greater information recall [34]. This is especially beneficial in a patient group that is at risk of poor information recall due to the age-related effects on cognitive function [35]. Preparatory information could potentially also help patients to more fully participate in decision making during a visit [36]. Using a patient portal to deliver information on individual fall-risk and fall-prevention information to older adults would reach approximately 60% of older fallers according to our data, which is in line with previous data showing that 71% of older adults have at least one patient portal account [37]. Despite more than half of our participants indicating that they would use a patient portal, not all older adults with a lived fall experience will be reached if a patient portal were the only method used to transmit fall-related information. Therefore, further research is needed to explore how to effectively convey fall-related information to the approximately 40% of participants who do not wish to use a patient portal.

### Barriers

Successful uptake of the proposed portal is dependent on an understanding of the barriers older fallers perceive with regards to using a portal. Concern about falling and the lack of relevance of the information were the most important reasons for patients not wanting to know their fall risk and consequently, not wanting to use a portal containing fall-risk information. Our study confirms that barriers to portal use previously identified in other studies are also applicable to older adults with lived fall experience [19]. We also identified privacy concerns and a portal that is not perceived beneficial to a patient’s health as important barriers. Moreover, the preference for real-life contact with a doctor rather than a digital tool was another important reason for not wanting to use a fall-prevention patient portal.

### Facilitators

In line with previous research [19], we found that a physician recommendation was a facilitator in all European regions. As suggested by our participants, inviting formal and informal caregivers to assist older fallers in using the patient portal could facilitate the use of the patient portal. This concurs with previous studies, in which informal caregivers also expressed a high interest in gaining patient portal access to help them improve care for their relative [38]. However, giving informal caregivers access to a patient’s personal health information can also pose privacy and security issues which would need to be addressed and securely managed [39].

### Regional differences

When comparing different regions we found that participants from Northern Europe were least inclined to use a patient portal for fall-risk information. This region also reported a lack of internet access and unfamiliarity with computers as important barriers which might be the explanation for the low level of intention to use a patient portal. Previous studies show that around 50% of adults 65 years and over have internet access in Northern Europe [40, 41]. This is higher than what we found, but the higher age of our participants may explain this [42].

A striking regional difference in facilitating factors was that the recommendation by a family member was most prominent among participants from Southern and Eastern Europe, where the family plays an important role in the culture [43].

Differences in information preferences were most clear for Eastern European participants. They wanted more information on falls and fall-prevention measures, their other health conditions and experiences of others compared to other regions. Moreover, they were among the highest regions for wanting to know their fall risk.

These results once again underscore the critical significance of UCD, with particular emphasis on usability research, in overcoming the barriers found in our study.

### Strengths and limitations

This European survey study has several strengths. It included participants from seven European countries, the participant population was diverse with regard to age, education level, living situation, and general health, thus increasing the generalizability of our findings. We also explored European regional differences in patient portal preferences.

On the other hand, our study also has several limitations. Due to the small sample size, the numbers of individuals recruited per region were also relatively small. Also, 15 participants reported difficulties filling in the questionnaire because they had no experience with digital health technology. This resulted in patients not completing the questionnaire or skipping questions. The lack of experience with digital health technology could have made it difficult for a participant to imagine what content features they preferred or what barriers or facilitators they perceived with regards to a patient portal. Visual examples of a portal might have better allowed patients to express their wishes and concerns for a patient portal.

Our survey was translated into six languages enabling a broad inclusion of European nations. Though translated by native speakers, no backward translation was performed thus slight inaccuracies in the translations cannot be ruled out.

### Future work

The preferences, barriers, and facilitators identified by this and similar previous studies [19] could be used to inform future fall-prevention patient portal development. Because of the small sample size, this study can be considered as a first step. Our survey should be repeated in a larger study with more participants over all European regions. In the meantime, we will continue with the AD*F*ICE_IT project and use the results of this study to develop a medication-related fall patient portal, since the studied population closely reflects the end-users of the AD*F*ICE_IT portal. Moreover, we will test the portal’s usability to make sure it fits with the preferences of the end-users. After completion, a trial will be performed to explore if using a clinical decision support system and patient portal increases shared decision-making on fall prevention measures and if patients using a portal better adhere to treatment advice compared to those receiving standard care, and ultimately if there is an effect on fall prevention [24].

Given that not all participants could nor would want to use a patient portal and that the expressed need for fall-prevention information was not affected by internet access, alternative routes to provide this information might also be helpfully explored in future studies. Ways to enable caregivers to also have access to the patient portal in a secure and acceptable way might also be helpful to explore in future studies.

## Conclusion

In this survey conducted in seven European countries, older adults with lived falls experience expressed a clear wish for fall-prevention information. Providing such information digitally via a patient portal appears to be a promising approach for future intervention studies and clinical practice. Though previous experience of using a patient portal was limited, the majority of participants expressed a wish to use a fall-prevention patient portal if available. Lack of computer skills and/or internet access appeared to be important barriers for usage, as well as privacy issues and payment for usage. Ensuring user-friendliness of the portal and recommendation by the treating physician seemed to be facilitating factors for use of the patient portal. However, as our results implied that a patient portal will not be used by all, alternative, non-digital solutions to inform and educate patients remain important. Therefore, a dual approach may be the preferred way to provide optimal fall prevention information and advice in both clinical practice and future trials.

### Supplementary Information

Below is the link to the electronic supplementary material.

Supplementary file1 (DOCX 49 KB)

Supplementary file2 (DOCX 26 KB)

Supplementary file3 (DOCX 17 KB)

Supplementary file4 (DOCX 19 KB)

## Data Availability

Data is available on request.
