# Supplementary material for: A European survey of older peoples’ preferences, and perceived barriers and facilitators to inform development of a medication-related fall-prevention patient portal
Source: Eur Geriatr Med. 2024 Apr 8;15(3):817–29. doi: 10.1007/s41999-024-00951-w (PMC11329398; doi:10.1007/s41999-024-00951-w)
Supplement: Supplementary file 1 — Supplementary file1 (DOCX 49 KB) [file 41999_2024_951_MOESM1_ESM.docx]

**Supplement 1: Survey, English version**

**Communicating medication-related fall risk with patients: International patient survey**

Dear Sir/Madam,

Falls are a major health problem in older adults. Falls often lead to injuries and are responsible for a decrease in mobility, independence and quality of life.

For the purpose of this study, entitled “Communicating medication-related falls-risk with patients” we would like to invite you to answer various questions about yourself, your medical background and about the information sources you use to obtain medical information. We are particularly interested in exploring ways in which patients can be enabled to use the Internet to find helpful health information and to increase their knowledge of their own condition and their medication.

Your contribution is of utmost importance to us. Thank you in advance for participating. In total this survey will take approximately 10 to 15 minutes.

This questionnaire can be filled in by people that are:

1. 65 years or older

and

1. who have sought medical advice (for example consulted their GP or a hospital specialist or who have attended hospital) due to a fall (or falls) or fall-related injury within the past year.

This survey is being conducted in 12 European countries. The coordinating center for the study is the Geriatric Medicine Department of the Academic Medical Centre in Amsterdam.

The survey was approved by the Medical Ethics Committee of the Academic Medical Centre and is performed under the responsibility of the Academic Medical Center in Amsterdam. You are therefore guaranteed that:

1. Your personal information and data will be processed anonymously and will not be distributed to third parties or traceable in any publications resulting from this survey.
2. Participation occurs voluntarily, meaning that you can withdraw from participation at any time before or during the survey without providing any reason. Withdrawal will not affect your treatment.
3. Participation in this study will not do you any harm and there is no risk concerning your wellbeing.

We hope that we have given you sufficient information about the survey in this letter. However, in case you might have any remarks or complaints about the process used or procedure followed, please contact he local Representative for this study: Yvonne Morrissey, Department of Health Care of Older People, Kent and Canterbury Hospital, Ethelbert Road, Canterbury, CT1 3NG. Telephone No 01227 766877 Extension 722 4208

We look forward to your participation in this survey.

Yours sincerely,

Dr. Yvonne Morrissey

Kim Ploegmakers, MSc

Dr. Annemiek Linn

Dr. Nathalie van de Velde

Prof. Julia van Weert

Before proceeding please confirm that you have read the Informed Consent form on the previous page and tick the box if you agree with these terms.

I have read and understood the information on the previous page about the “Communicating medication-related falls risk with patients” International Patient Survey and I am willing to participate in this survey.

Yes

No → Unfortunately, you cannot participate in this online survey if you do not give Informed Consent

**Demographics**

The survey starts with some general question about your personal circumstances.

1. In which country do you live?

Belgium

Czech Republic

Denmark

Spain

France

Ireland

Italy

Malta

The Netherlands

Austria

Poland

Finland

Sweden

Turkey

United Kingdom

1. Are you 65 years or older?

Yes

No → Unfortunately you cannot participate if you are not 65 years or older

1. What is your age?

1. What is your gender?

Male

Female

1. What is the highest level of education you have completed?

Nursery school/ Pre-school up to age 4

Primary school education/ Infant school/ Junior school

Lower Secondary school education - usually including CSE/ O levels

Upper Secondary school education (or “sixth form” level) usually including A levels

Vocational training, vocational qualifications or Apprenticeship

University and Polytechnic Education usually leading to Bachelor’s degree

Masters degree, Post-graduate diplomas and certificates

PhD, Doctorate

1. What is your current living situation?

Living at home independently

Living at home but receiving help regularly*

Living in a nursing home

*Receiving help: for example with regard to personal hygiene, cooking and cleaning or grocery shopping.

**Medical information**

We will now ask you some questions about your health conditions and any recent consultations you may have had with your GP and/or Hospital Specialist regarding a fall.

When we talk of a fall we mean, “An unexpected event in which you came to rest on the ground, the floor or any lower level.” A fall can occur with or without sustaining injury.

1. Have you consulted your GP or a Hospital Specialist or attended hospital due to a fall or fall-related injury during the past year?

Yes

No → Unfortunately, you cannot participate in this online survey. Only people who have experience a fall or fall related injuries and have consulted their GP or Hospital Specialist for it can participate.

1. How many times have you fallen in the previous year?

Once

Two or more times

Before we continue with questions, here is some information for you:

Medications are often very effective for treating diseases. However we are also aware some medications can have unwanted effects, for instance they can increase the risk of falling.

In this survey we wish to find out how much is known by patients about the falls-risk that may be associated with certain medications or combinations of medications.

1. Are you aware that certain medications can increase your Falls-risk as a side effect?

Yes → go to question 4

No → go to question 5

1. How did you learn that medications can cause a fall?

My doctor told me

Someone else told me

I read it on a leaflet/ in a newspaper/ brochure/ magazine

I read it on the Internet

Other, please write any other information source in the box below:

1. Did your doctor suggest replacing, stopping or changing the dosages of any of your medications in order to reduce the risk of a further fall in the future?

No, it was not discussed

Yes, we discussed this, but it was not necessary

Yes, (some) medications were stopped, reduced or changed

Yes, but I didn’t agree with it

I don’t remember

Other, namely

**Patient portals**

The next set of questions is about “Patient Portals”. Currently, Patient Portals are used in many hospitals internationally.

A Patient Portal is an individual, secured computer website with personal information for patients (e.g. Appointments, Lab test results etc.). A Patient Portal is an easy way to manage your health information at any location (e.g. at home) at a time that’s convenient to you. Additionally, it has the potential to allow you to ask your health care provider questions by email.

1. Do you have easy access to the Internet?

No, I don’t have access to Internet

Yes, I have access but I can only use it with help of others

Yes, but not in my own home

Yes, I have Internet access at home

1. Have you ever used a Patient Portal that provided you information about your health conditions?

Yes

No

1. Would you use a Patient Portal that gave you access to your medical record, test results and other features described above?

No → go to question 4

Maybe → go to question 4

Yes → go to question 5

1. Why would you **not** use a Patient Portal (you can tick one or more boxes)?

I have no access to the Internet

I have difficulty using Internet applications

I prefer to discuss my medical situation with my doctor

Other, please write any other reasons in the box below:

We want to develop a Patient Portal that not only provides you with information about your illness (and all other features we described above) but also provides you with information about your personal Falls-risk. You would be able to check each individual medication in your prescription to see if this medication increases your falls-risk or not. Imagine you have access to such a Portal and then please answer the following questions:

1. Would you like to know your Falls-risk estimation per medication?

No → go to question 6

Maybe → go to question 6

Yes → go to question 7

1. Why don’t you want to know your Falls-risk estimation per drug class (you can tick one or more boxes)?

It’s not relevant for me, I have never fallen because of my medication

I am not interested in my Falls-risk

It scares me to know my Falls-risk

Other, please write any other reason in the box below:

1. What other elements should the Patient Portal contain? (you can tick one or more boxes)

Information about my illness

Information about falls

Information about ways of managing Falls-related conditions like dizziness or low blood pressure

Information about medications that can increase Falls-risk

Information about medication interactions (how different medication can influence each other)

Information on how I can prevent falls

Information on how I can improve my health in general

Physical exercises I can do to stay healthy

Physical exercises I can do to help prevent falls

Experiences of other patients with the same illness as mine

Ability to print information that you can find on the patient portal

Useful links to other health websites

Access to my medical record

Access to my test results

Access to my hospital discharge letter/ clinic letter

Email communication with my physician

Order a repeat prescription

Reminder about an appointment

Dictionary of medical jargon

Other, please write any other element in the box below

1. What would you do with your personal Falls-risk estimation? (you can tick one or more boxes)

Nothing

I would discuss it with my GP and/or Hospital specialist

I would look up more information about falls, for example on the Internet

I would look up information on how to prevent a fall

Other, please write any other reason in the box below:

**Barriers and Facilitators**

Previous experience has taught us that the way a Patient Portal is designed can encourage or put people off using it. We want to know to what extent factors in the design might apply to your personal situation.

1. Below you will find different barriers that might put you off using the portal. Please select the **four barriers** that apply to you most.

It would put me off using the Portal:

If the use of the Patient Portal doesn’t improve my health

If the Patient Portal is only available online

If my privacy is not secured

If I have to pay for using the Patient Portal

If I don’t receive a fast response from my GP or Specialist in the message system

If I can’t communicate with my GP or Specialist in the messaging system

If it would be difficult to enter text information

If illustrations are too difficult to comprehend

1. Are there other factors that make you not use the patient portal that we didn’t mentioned above? Please fill in box below:

1. Below you will find different facilitators that might stimulate you to use the portal. Please select the **five facilitators** that apply to you most.

The support of a Specialist nurse would encourage me to use the Patient Portal

A video explaining the Portal would encourage me to use the Patient Portal

The recommendation of my GP or Specialist to use the Patient Portal would encourage me to use the Patient Portal

A recommendation of my family to use the Patient Portal would encourage me to use the Patient Portal

If the Patient Portal is easy to use, it is more likely that I will use the portal

If the information is easy to find, it is more likely that I will use the Portal

If I can share my personal medical information with my GP or Hospital specialist, it is more likely that I will use the portal

If the Patient Portal provides additional background information about my medical condition and health, it is more likely that I will use the Portal

If the Portal contains written information that is accompanied by illustrations, it is more likely that I will use the Portal

If the Patient Portal takes voice commands (e.g. give a voice command instead of typing the command) it is more likely that I will use the Portal

1. Are there other factors that make you use the patient portal that we didn’t mentioned above? Please fill in box below:

1. Please indicate to what extent you agree with the following statements
2. I feel confident that I can use a Patient Portal

| Not applicable to me at all | Not applicable to me | Neutral | Applicable to me | Strongly applicable to me |
| --- | --- | --- | --- | --- |
|  |  |  |  |  |

1. I am anxious about using computers

| Not applicable to me at all | Not applicable to me | Neutral | Applicable to me | Strongly applicable to me |
| --- | --- | --- | --- | --- |
|  |  |  |  |  |

1. I am familiar with using computers

| Not applicable to me at all | Not applicable to me | Neutral | Applicable to me | Strongly applicable to me |
| --- | --- | --- | --- | --- |
|  |  |  |  |  |

1. I never use the internet for health-related purposes

| Not applicable to me at all | Not applicable to me | Neutral | Applicable to me | Strongly applicable to me |
| --- | --- | --- | --- | --- |
|  |  |  |  |  |

1. Generally, I have difficulty finding or using websites

| Not applicable to me at all | Not applicable to me | Neutral | Applicable to me | Strongly applicable to me |
| --- | --- | --- | --- | --- |
|  |  |  |  |  |

1. Generally, I have difficulty remembering usernames and passwords

| Not applicable to me at all | Not applicable to me | Neutral | Applicable to me | Strongly applicable to me |
| --- | --- | --- | --- | --- |
|  |  |  |  |  |

1. I have physical limitations that prevent me from using the computer or the Internet (e.g. visual or hearing impairments or mobility impairments)

| Not applicable to me at all | Not applicable to me | Neutral | Applicable to me | Strongly applicable to me |
| --- | --- | --- | --- | --- |
|  |  |  |  |  |

1. I would need help of others to use the computer or the Internet

| Not applicable to me at all | Not applicable to me | Neutral | Applicable to me | Strongly applicable to me |
| --- | --- | --- | --- | --- |
|  |  |  |  |  |

**General health**

These questions refer to your health and medical conditions in the ***last month***.

1. Do you use 5 or more different types of medication?

Yes

No

I don’t know

1. Are you using a walking aid? (you can tick one or more boxes)

No

Yes, a stick

Yes, a walker

Yes, a wheelchair

Yes, a mobility scooter or an electric wheelchair

1. How would you rate your **overall health** during the last month on a scale from 1 to 10, if 1 is very bad and 10 is very good?

| 1 | 2 | 3 | 4 | 5 | 6 | 7 | 8 | 9 | 10 |
| --- | --- | --- | --- | --- | --- | --- | --- | --- | --- |
|  |  |  |  |  |  |  |  |  |  |

Thank you for your participation in this online survey

It is important that you always consult your GP, specialist or pharmacist if you want to stop usage or reduce dosage of your medication. Tell your doctor or pharmacist if you are experiencing side effects. Perhaps you can use less of this medication or another drug is more suitable for you.
