# Supplementary material for: A European survey of older peoples’ preferences, and perceived barriers and facilitators to inform development of a medication-related fall-prevention patient portal
Source: Eur Geriatr Med. 2024 Apr 8;15(3):817–29. doi: 10.1007/s41999-024-00951-w (PMC11329398; doi:10.1007/s41999-024-00951-w)
Supplement: Supplementary file 2 — Supplementary file2 (DOCX 26 KB) [file 41999_2024_951_MOESM2_ESM.docx]

**Supplement 2: Overview of origin barriers and facilitators**

| Barriers originated from Sakaguchi-Tang et al [1] | Barriers originated from Technology Acceptance Model (TAM) [2] |
| --- | --- |
| Paying for usage | Paying for usage |
| Privacy issues | Doesn’t improve my health |
| Doesn’t improve my health |  |
| Only available online |  |
| Slow response from doctor |  |
| Can’t communicate with doctor |  |
| Difficult to enter text |  |
| Illustrations difficult to comprehend |  |

| Facilitators originated from Sakaguchi-Tang et al [1] | Facilitators originated from Technology Acceptance Model (TAM) [2] |
| --- | --- |
| Easy to use | Easy to use |
| Easy to find information | Easy to find information |
| Use recommended by my doctor | Use recommended by my doctor |
| Share my medical information with my doctor | Use recommended by my family |
| Support from nurse |  |
| Additional info on my illness and health |  |
| Video explaining portal |  |
| Use recommended by my family |  |
| Voice commands |  |
| Written info accompanied by illustrations |  |

[1] Sakaguchi-Tang DK, Bosold AL, Choi YK, Turner AM. Patient portal use and experience among older adults: Systematic review. JMIR Med Informatics 2017;5. https://doi.org/10.2196/medinform.8092.

[2] Davis FD. Perceived usefulness, perceived ease of use, and user acceptance of information technology. MIS Q Manag Inf Syst 1989;13:319–39. https://doi.org/10.2307/249008.
