# Supplementary material for: A European survey of older peoples’ preferences, and perceived barriers and facilitators to inform development of a medication-related fall-prevention patient portal
Source: Eur Geriatr Med. 2024 Apr 8;15(3):817–29. doi: 10.1007/s41999-024-00951-w (PMC11329398; doi:10.1007/s41999-024-00951-w)
Supplement: Supplementary file 3 — Supplementary file3 (DOCX 17 KB) [file 41999_2024_951_MOESM3_ESM.docx]

**Supplement 3:** Relation between patient portal experience and intent to use based on internet access (n=119).

|  | Internet at home (n=56; 46.7%) | Internet with help (n=22; 18.5%)^¶^ | Internet but not at home  (n=2; 1.7%) | No internet access  (n=39; 32.5%) |
| --- | --- | --- | --- | --- |
| Experience patient portal (yes) | 46.4%* | 4.5% | 0 | 2.6%* |
| Use patient portal  Yes  No  Maybe | 70.9%*  16.4%*  12.7% | 40.9%  45.5%  13.6% | 0  50%  50% | 16.2%*  73.0%*  10.8% |
| Know fall risk  Yes  No  Maybe | 80.4%  16.1%  3.6% | 72.7%  9.1%  18.2% | 0  100%  0 | 54.1%  37.8%  8.1% |

^¶^Compared to table 1 of the manuscript, one participant was excluded from this analysis due to missing data. *: p< 0.05.
