# Supplementary material for: A European survey of older peoples’ preferences, and perceived barriers and facilitators to inform development of a medication-related fall-prevention patient portal
Source: Eur Geriatr Med. 2024 Apr 8;15(3):817–29. doi: 10.1007/s41999-024-00951-w (PMC11329398; doi:10.1007/s41999-024-00951-w)
Supplement: Supplementary file 4 — Supplementary file4 (DOCX 19 KB) [file 41999_2024_951_MOESM4_ESM.docx]

**Supplement 4** Number of selected features, barriers, and facilitators in Europe and per region

**Number of selected features by participants in Europe and per region.**

| Number of features selected | Europe (n=122) | North (n=29) | West (n=42) | South (n=20) | East (n=31) |
| --- | --- | --- | --- | --- | --- |
| 0 | 10.7% | 31.0% | 7.3% | 5.0% | 0.0% |
| 1 | 7.4% | 6.9% | 7.3% | 0.0% | 12.9% |
| 2 | 5.0% | 3.4% | 7.3% | 0.0% | 6.5% |
| 3 | 4.1% | 3.4% | 7.3% | 0.0% | 3.2% |
| 4 | 9.9% | 10.3% | 2.4% | 25.0% | 9.7% |
| 5 | 14.9% | 10.3% | 12.2% | 45.0% | 3.2% |
| 6 | 5.0% | 3.4% | 2.4% | 15.0% | 3.2% |
| 7 | 5.0% | 10.3% | 4.9% | 0.0% | 3.2% |
| 8 | 5.8% | 3.4% | 12.2% | 0.0% | 3.2% |
| 9 | 3.3% | 3.4% | 4.9% | 0.0% | 3.2% |
| 10 | 3.3% | 3.4% | 2.4% | 0.0% | 6.5% |
| 11 | 2.5% | 0.0% | 4.9% | 5.0% | 0.0% |
| 12 | 1.7% | 0.0% | 4.9% | 0.0% | 0.0% |
| 13 | 1.7% | 0.0% | 4.9% | 0.0% | 0.0% |
| 14 | 4.1% | 0.0% | 9.8% | 0.0% | 3.2% |
| 15 | 1.7% | 0.0% | 0.0% | 0.0% | 6.5% |
| 16 | 4.1% | 0.0% | 2.4% | 5.0% | 9.7% |
| 17 | 1.7% | 0.0% | 0.0% | 0.0% | 6.5% |
| 18 | 4.1% | 0.0% | 2.4% | 0.0% | 12.9% |
| 19 | 1.7% | 0.0% | 0.0% | 0.0% | 6.5% |
| 20 | 2.5% | 10.3% | 0.0% | 0.0% | 0.0% |

n: number of participants. Northern Europe: Denmark and the United Kingdom; Western Europe: the Netherlands; Southern Europe: Italy and Spain; Eastern Europe: Czech Republic and Türkiye.

**Number of selected barriers by participants in Europe and per region.**

| Number of barriers selected | Europe (n=122) | North (n=29) | West (n=42) | South (n=20) | East (n=31) |
| --- | --- | --- | --- | --- | --- |
| 0 | 15.7% | 34.5% | 12.2% | 5.0% | 9.7% |
| 1 | 14.9% | 17.2% | 19.5% | 5.0% | 12.9% |
| 2 | 14.9% | 13.8% | 19.5% | 10.0% | 12.9% |
| 3 | 13.2% | 13.8% | 17.1% | 5.0% | 12.9% |
| 4 | 38.0% | 20.8% | 31.7% | 75.0% | 38.7% |
| 5 | 2.5% | 0 | 0 | 0 | 9.7% |
| 7 | 0.8% | 0 | 0 | 0 | 3.2% |

n: number of participants. Northern Europe: Denmark and the United Kingdom; Western Europe: the Netherlands; Southern Europe: Italy and Spain; Eastern Europe: Czech Republic and Türkiye.

**Number of selected facilitators by participants in Europe and per region.**

| Number of facilitators selected | Europe (n=122) | North (n=29) | West (n=42) | South (n=20) | East (n=31) |
| --- | --- | --- | --- | --- | --- |
| 0 | 25 (20.7%) | 48.3% | 14.6% | 5.0% | 12.9% |
| 1 | 14 (11.6%) | 20.7% | 9.8% | 5.0% | 9.7% |
| 2 | 9 (7.4%) | 3.4% | 14.6% | 0 | 6.5% |
| 3 | 15 (12.4%) | 0 | 26.8% | 5.0% | 9.7% |
| 4 | 31 (25.5%) | 6.9% | 14.6% | 75.0% | 25.8% |
| 5 | 21 (17.4%) | 20.7% | 17.1% | 10.0% | 19.4% |
| 6 | 1 (0.8%) | 0 | 2.4% | 0 | 0 |
| 9 | 2 (1.7%) | 0 | 0 | 0 | 6.5% |
| 10 | 3 (2.5%) | 0 | 0 | 0 | 9.7% |

n: number of participants. Northern Europe: Denmark and the United Kingdom; Western Europe: the Netherlands; Southern Europe: Italy and Spain; Eastern Europe: Czech Republic and Türkiye.
